# Synchrotron X-ray Fluorescence Microscopy Reveals Trace Elemental Indicators of Life History in Marsupial Teeth

**DOI:** 10.1007/s12011-024-04502-z

**Published:** 2025-01-16

**Authors:** William M. G. Parker, Justin W. Adams, David P. Hocking, Erich M. G. Fitzgerald, Geoff Shaw, Marilyn B. Renfree, Alistair R. Evans

**Affiliations:** 1 School of Biological Sciences, Monash University, Melbourne, VIC 3800 Australia; 2 Museums Victoria Research Institute, Museums Victoria, Melbourne, VIC 3001 Australia; 3 Biomedicine Discovery Institute, Monash University, Melbourne, VIC 3800 Australia; 4 Vertebrate Zoology and Palaeontology, Tasmanian Museum and Art Gallery, Hobart, TAS 7001 Australia; 5 School of BioSciences, The University of Melbourne, Melbourne, VIC 3010 Australia

## Abstract

As teeth develop, their mineralised composition is a bio-recorder of diet, environment, and growth. High-resolution elemental mapping provides a tool to reveal records of life history within teeth. The relative concentrations of a range of trace elements change between in utero development, birth, and weaning in eutherian mammals. Marsupials, however, have a different mode of development: altricial birth and growth within the pouch facilitated by compositional transitions in milk. How these differences alter patterns of elemental mineralisation and become recorded in marsupial teeth is previously unknown. This study analyses the distribution of calcium (major element), zinc (actively incorporated trace element), and strontium (passively incorporated trace element) in the teeth of five species of diprotodontian marsupial using synchrotron X-ray fluorescence microscopy. We find that the diprotodontian lower incisor concatenates elemental variation from across the molariform dentition, preserving a prolonged record of life history in four of the five species. Patterns of elemental incorporation in enamel, dentine, and cementum are presented, with Ca, Zn, and Sr having differing distributions. Zn accretion indicates a role in mineralisation and/or prevention of tooth degradation. Zn also demarcates incremental cementum lines. Sr is shown to be passively incorporated into marsupial teeth, with increasing Sr concentration in milk recorded in dental tissues formed contemporaneously. Older individuals have oscillatory signals in Sr that appear linked to seasonality. These findings highlight some similarities between eutherian and marsupial trace element incorporation, particularly in the distribution of Zn. Sr signals in marsupial teeth record key aspects of life history.

**Supplementary Information:**

The online version contains supplementary material available at 10.1007/s12011-024-04502-z.

## Introduction

Teeth can provide a wealth of information about an animal’s biology. Tooth growth, morphology, wear, breakage, and composition all give clues to lifespan, development, feeding mode, diet, and environment [1–5]. When investigating the early life history and development of living and extinct animals, trace element incorporation within dental tissues, particularly when measured at high resolution, is an extremely powerful tool [6, 7]. High-resolution trace elemental mapping of teeth via either Laser Ablation Inductively Coupled Plasma Mass Spectrometry (LA-ICP-MS) or X-Ray Fluorescence Microscopy (XFM) has now been applied to a range of primates, producing biologically informative results [2, 6, 8–10]. Weaning, repeated bouts of breastfeeding, environmental contamination, and periodic illness have all been revealed using trace elemental records within the teeth of hominids [2, 6, 7, 11, 12]. Whilst we see more prominent applications in primatological and human archaeological contexts, understanding of trace element distribution in the teeth of other eutherian mammals is limited to far fewer taxa e.g. [13, 14]. Data for marsupials, a mammalian clade characterised by their mode of development, is even more scarce.

Marsupials are excellent candidates for the study of life history via trace elemental incorporation into teeth. In general, they are vastly understudied mammals with poor records of life history and ecology for many species. In a recent analysis of marsupial life-history continua, of the ~ 350 extant species, only 37 had sufficiently complete data for analysis [15]. This highlights the need for alternate ways to collect these life history data. A limited number of marsupial species have well-characterised life history data, and these provide opportunities to link tooth composition and formation with key developmental events [16, 17]. Also advantageous is that for some species within the Order Diprotodontia, the formation of the procumbent lower incisors (for which the group is named) continues for a highly extended period [5]. This should allow a prolonged record of life history to be collected from a single tooth without the need to concatenate elemental assays across the dentition.

This study reports on the first systematic elemental mapping of marsupial teeth, collecting fundamental data on trace element incorporation. These data will facilitate comparison of marsupial trace element uptake with trends seen in eutherian mammals [2, 12–14]. It should not be assumed that the composition of marsupial teeth will reflect similar patterns to those seen in eutherians. The developmental differences between marsupial and eutherian mammals mean that key events recorded in eutherian teeth are likely to be absent from marsupials altogether. Altricial birth with young developing in the pouch means that no in-utero record is expected to be seen in the teeth of marsupials, nor any record of the birth event, histologically identifiable as the neo-natal line in some eutherians [9, 14, 18]. This is evidenced by 3D tooth histology of the Tammar wallaby, *Notamacropus eugenii*, showing that mineralisation of the earliest forming tooth that is not resorbed, the procumbent lower incisor, only begins after birth [17]. Further to this, the composition of breast milk is relatively uniform in eutherian mammals through lactation, but in marsupials, composition alters markedly to accommodate the changing nutritional needs of the young as they develop within the pouch [19, 20]. In the Tammar wallaby, milk formed at the commencement of lactation has a dry matter concentration of 12% (w/w) [21]. When the young commences weaning, 36 weeks later, this solid fraction has risen to 40% (w/w) [21]. Compositional shifts in major and trace element composition are also evident in the milk of the brush-tailed possum (*Trichosurus vulpecula*) throughout pouch young development [22]. Whilst compositional analysis of milk has not been undertaken for the majority of marsupials, it is likely that some aspects of changing milk composition will be captured in the elemental record mineralised within enamel and dentine.

This study applies synchrotron XFM to assay a range of elements within the teeth of four genera (Tammar wallaby—*Notamacropus eugenii*, eastern grey kangaroo—*Macropus giganteus*, western grey kangaroo—*Macropus fuliginosus*, brush-tailed possum—*Trichosurus vulpecula*, and bare-nosed wombat—*Vombatus ursinus*) of diprotodontian marsupial.

## Methods

### Selection of Species for Sampling

The selected taxa encompass a range of body sizes and phylogenetic diversity within Diprotodontia (Table 1). *N. eugenii* is a model species for the study of marsupials and has very well-constrained development [16, 17]. *Macropus giganteus* and *M. fuliginosus* are, like *N. eugenii*, macropodids but represent larger body sizes. Average adult female masses are 4–6 kg for *N. eugenii* compared to 17–42 and 17–39 kg for *M. giganteus* and *M. fuliginosus*, respectively [26].

**Table 1:** Specimens examined as part of this study. All samples are modern and originate from south-eastern Australia, with the exception of the three individuals of *T. vulpecula* sampled from Tasmania, Australia

Species	Specimen number	Sampled tooth type	Institution	Age category and sex (if known)	Further details*
*Notamacropus eugenii*	7024	I_1_, M_1_, M_2_, M_3_, and M_4_	The University of Melbourne	Sub-adult Male	Born in captive colony Age = 22–24 months
	6623	I_1_	The University of Melbourne	Adult	Entered captive colony as adult
	7140	I_1_	The University of Melbourne	Adult	Entered captive colony as adult
*Macropus giganteus*	SC4317	I_1_	The University of Melbourne	Sub-adult Male	11 kg MI = 0.3 Age = 1 year
	SC4783	I_1_	The University of Melbourne	Adult Female	29 kg MI = 2.7 Age = 4.8 years
*Macropus fuliginosus*	GN11	I_1_	Monash University	Adult Female	MI = 3 Age = 5.9 years
*Trichosurus vulpecula*	TMAG A446	I_1_	Tasmanian Museum and Art Gallery	Adult Male	2.95 kg Age = 4–6 years
	TMAG A1088	I_1_	Tasmanian Museum and Art Gallery	Sub-adult Female	Age = 1.5–2.5 years
	TMAG A1173	I_1_	Tasmanian Museum and Art Gallery	Adult Male	Age = 4–6 years
*Vombatus ursinus*	NMV C22384	M^1^	Museums Victoria	Juvenile Female	11.6 cm skull length
	NMV C22381	M^1^	Museums Victoria	Sub-adult	14.2 cm skull length

*Ages of *Macropus* specimens were determined via the molar index (MI) equation described in [23]. Ages of *T. vulpecula* specimens were calculated using the wear state of the first upper molar as described in [24]. In the absence of species-specific data, *V. ursinus* age categories were assigned using measurements of skull length informed by [25]

*Trichosurus vulpecula* is phylogenetically distinct from the macropodids, as a member of Phalangeridae [26]. Currently, *T. vulpecula* is the only Australian marsupial in which milk trace element content has been quantified [22], making it an essential species for inclusion in this study. This species provides an important point of comparison to determine if maternal milk composition is directly reflected in contemporaneously growing teeth.

*Vombatus ursinus* (Vombatidae) represents a novel archetype due to its ever-growing incisors, premolars, and molars. In adult *V. ursinus*, it is expected that all of the mineralised juvenile records will be lost due to wear, with tooth composition reflecting adult diet [27]. As such, juvenile milk consumption and weaning is likely to only be recovered from the teeth of young individuals.

### Sample Preparation and Analysis

Teeth, representing five species, were obtained from the institutions listed in Table 1. Incisors, and in some cases, molariform teeth, were extracted from the jaw and embedded using Biodur E1 and E12A following the procedure described in [5].

Embedded molariform teeth were sectioned in the axiobuccolingual plane, *T. vulpecula* mandibular incisors were sectioned sagittally along the dorso-ventral axis, and macropodid mandibular incisors were sectioned in the dorsal plane (perpendicular to the dorso-ventral axis) using a water-cooled, diamond-tipped, stainless steel slow saw (Lortone, INC, Mukilteo, WA, USA). The sectioned surface was then polished using a 12-μm diamond grit, water-lubricated lapping machine (UNIPOL-820, MTI Corporation, Richmond, CA, USA) and 12-μm colloidal silicon carbide.

Specimens for XFM analysis had the polished surface of the sectioned tooth affixed to a standard glass histological slide using heated dental wax. The opposite side of this block was ground away using a petrographic grinding machine (Australian Petrographics, Queanbeyan, NSW, Australia) until a 300-μm wafer was left affixed to the slide. This section was released from the dental wax holding it to the slide, leaving a thin section of tooth maintained in a resin matrix. This section was mounted on an acrylic window so that an incident analytical beam, even if penetrating the full thickness of the sample, would only interact with tooth material. XFM analysis was undertaken on the X-ray fluorescence microscopy beamline using the Kirkpatrick-Baez microprobe in conjunction with the Maia detector at 18.5 keV at the Australian Synchrotron [28]. Aluminium foil shielding was employed to reduce the Ca counts reaching the detector which allowed an increase in flux to improve sensitivity to elements of interest (specifically Sr). Step sizes and scanning velocities were selected based on the area of the analysed tooth (Supplementary Table 1). Collected data were first deconvoluted and analysed in GeoPIXE [29] with subsequent analysis and visualisation in FIJI [30].

## Results

### XFM Elemental Mapping

XFM elemental maps were produced for Ca, Zn, and Sr for each sectioned tooth. Across the marsupials examined in this study, excluding the hypsodont dentition of *V. ursinus*, the fundamental behaviour of each element within each dental tissue (enamel, dentine, and cementum) showed similar trends.

### *Notamacropus eugenii*

**Fig. 1:**
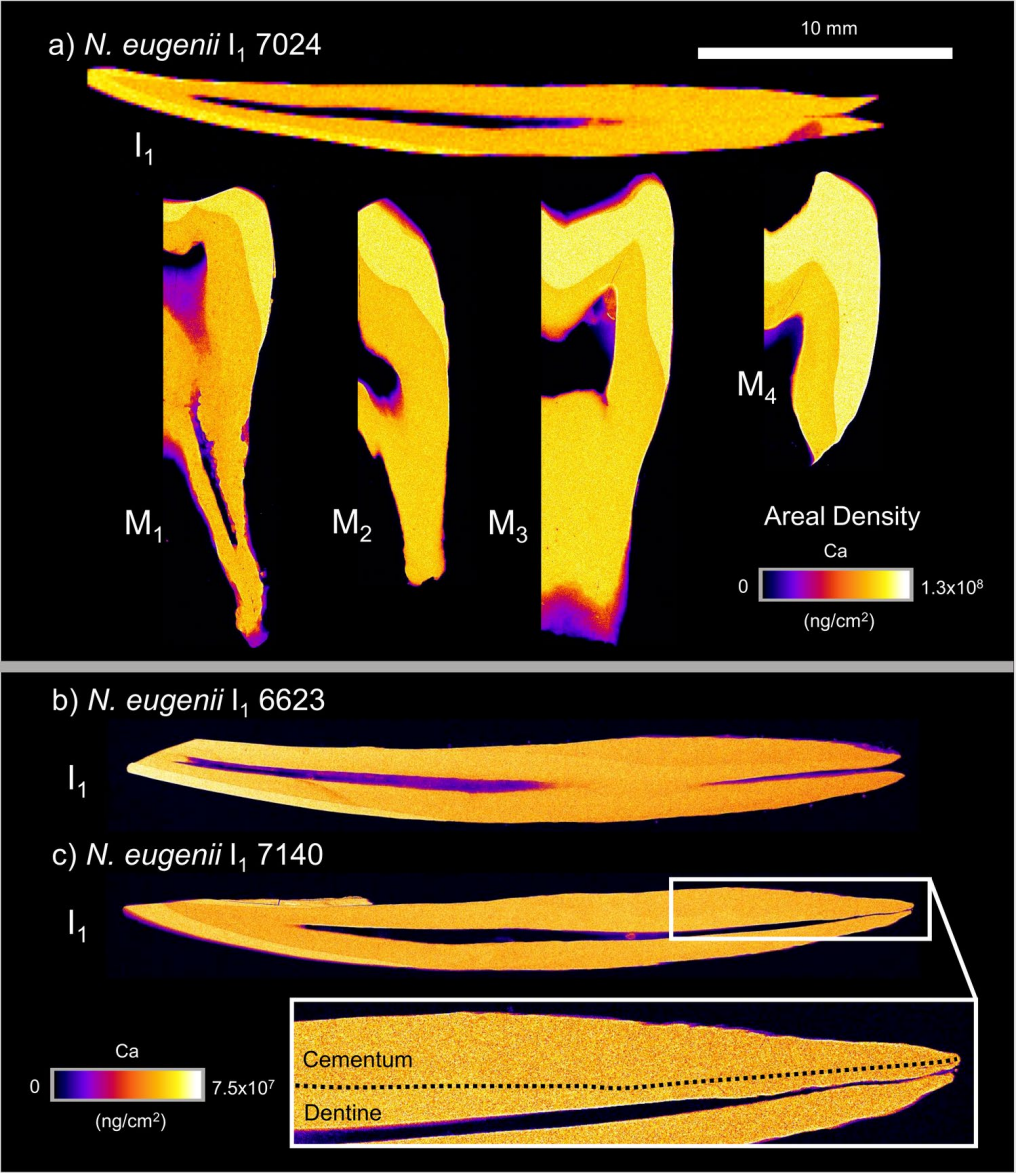
XFM maps of calcium areal density (ng/cm^2^) in sectioned teeth of three *N. eugenii* individuals (7024, 6623, and 7140). I_1_ is presented for all three individuals, with M_1–4_ also presented for individual 7024. Areal densities range from 0 to 1.3 × 10^8^ ng/cm^2^ for individual 7024 and from 0 to 7.5 × 10^7^ ng/cm^2^ for individuals 6623 and 7140. Inset region of 7140 shows the distribution of dentine and cementum within the incisor root. 10 mm scale bar is applicable to all teeth

Calcium abundance (Fig. 1) is uniformly highest within enamel and did not show systematic variation from the enamel-dentine junction to the outer enamel surface nor from the cuspal to the cervical enamel. Ca abundance in dentine and cementum is similar, being uniformly lower than enamel, and XFM mapping of Ca alone does not necessarily distinguish between these tissues (Fig. 1b, c). Slight variation is observed in Ca areal density within the enamel and dentine of each tooth (Fig. 1). Exceptions to this include exogeneous contamination of the root of 7024 I_1_ and at the edges of tooth sections where tooth thickness decreases and the incident beam may not be perpendicular to the tooth surface.

**Fig. 2:**
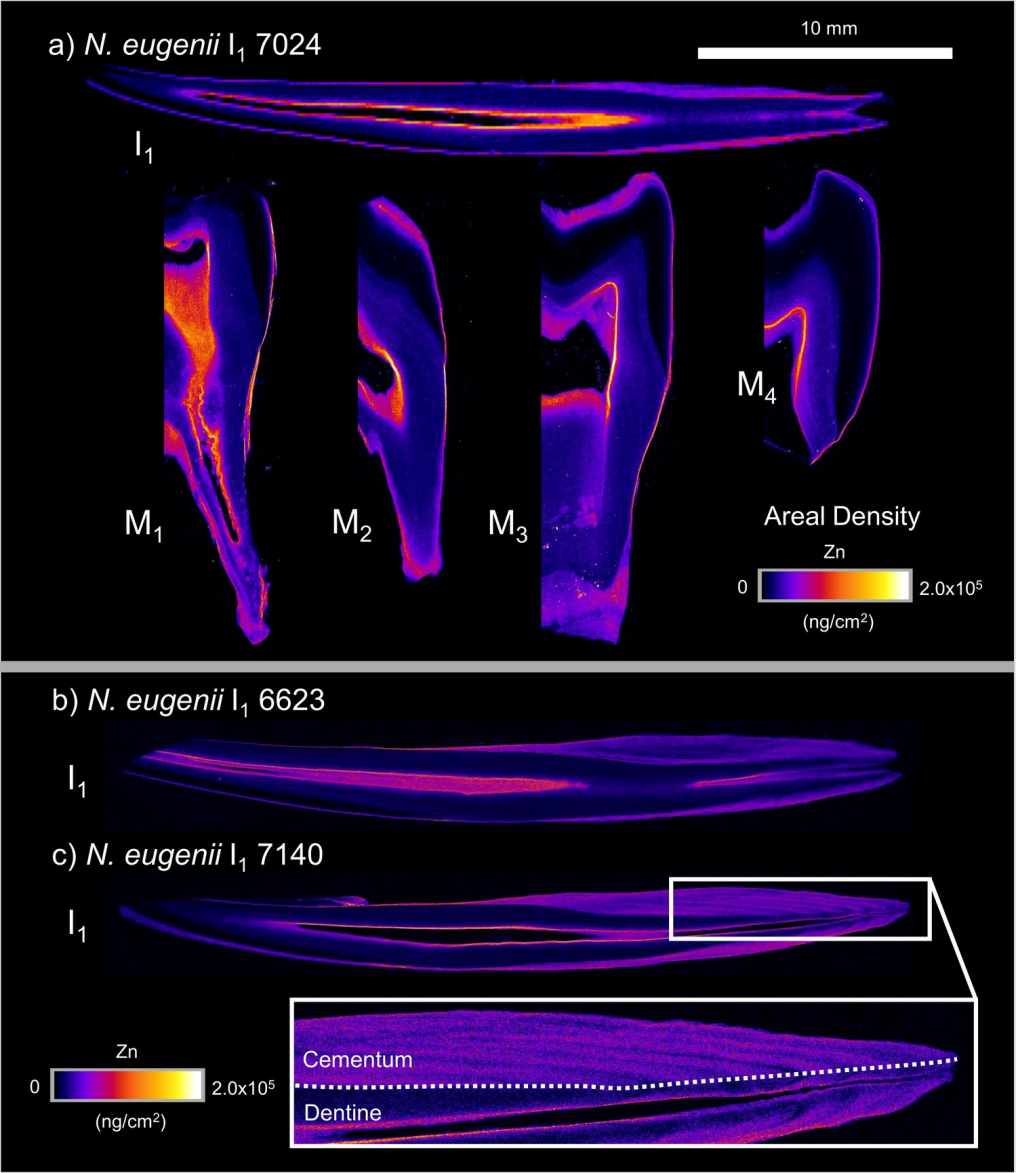
XFM maps of Zinc areal density (ng/cm^2^) in sectioned teeth of three *N. eugenii* individuals (7024, 6623, and 7140). I_1_ is presented for all three individuals, with M_1–4_ also presented for individual 7024. Areal densities range from 0 to 2.0 × 10^5^ ng/cm^2^ for all individuals. Inset region of 7140 shows the distribution of dentine and cementum within the incisor root. 10 mm scale bar is applicable to all teeth

Zn mapping (Fig. 2) reveals elevated density within the outer enamel, dentine adjacent to the pulp cavity and in cementum. Zinc abundance within enamel is highest at the outer enamel surface (~ 8.5 × 10^4^ ng/cm^2^, Fig. 2—M_3_) and decreases towards the enamel-dentine junction. Zn is higher in abundance in cementum (~ 1.3 × 10^5^ ng/cm^2^, Fig. 2—M_3_) and in regions of dentine exposed to the pulp cavity (~ 2.4 × 10^5^ ng/cm^2^, Fig. 2—M_3_) than in the majority of primary dentine (~ 1.8 × 10^4^ ng/cm^2^, Fig. 2—M_3_). Specimens 6623 and 7140 (Fig. 2b, c) show heterogeneity in Zn that demarcates sequential cementum layers towards the base of the root.

**Fig. 3:**
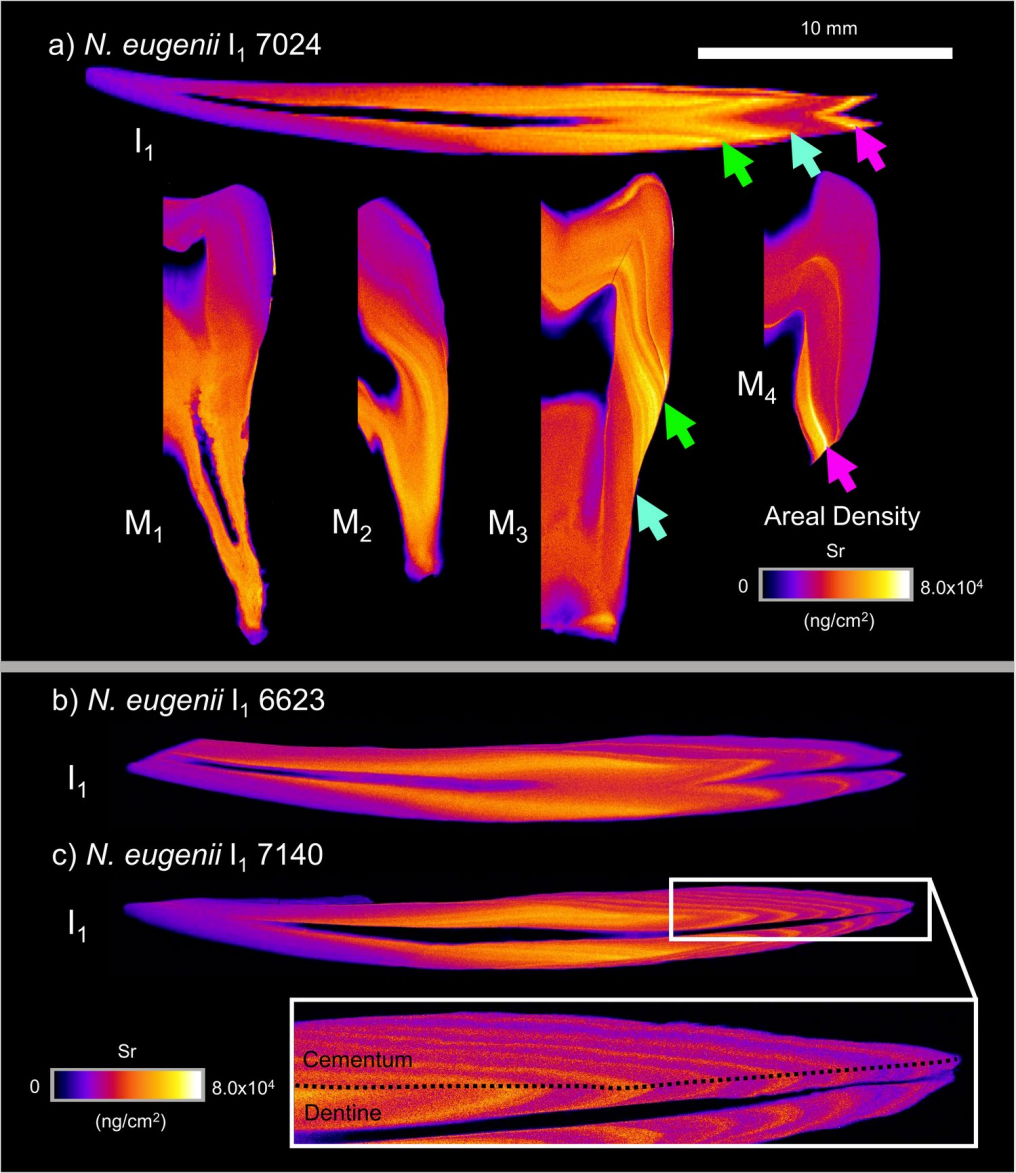
XFM maps of strontium areal density (ng/cm^2^) in sectioned teeth of three *N. eugenii* individuals (7024, 6623, and 7140). I_1_ is presented for all three individuals, with M_1–4_ also presented for individual 7024. Areal densities range from 0 to 8.0 × 10^4^ ng/cm^2^ for all individuals. Arrows highlight compositional transitions seen in molar teeth and the incisor. Green arrow—local minimum in Sr areal density, blue arrow—transitional decrease, pink arrow—transitional increase. Inset region of 7140 shows the distribution of dentine and cementum within the incisor root. 10 mm scale bar is applicable to all teeth

Strontium incorporation (Fig. 3) shows limited association with the tissue type (enamel, dentine, or cementum) being assayed. Instead, timing of tissue mineralisation appears to be the main factor contributing to Sr abundance. This is evidenced by similar Sr areal density across enamel and dentine that were deposited at the same time, with this also being the case for dentine and cementum. This pattern can be particularly well visualised in the incisor root displayed in Fig. 3c, inset where the apex of the chevron in Sr abundance on either side of the pulp cavity marks the boundary between exterior cementum and interior dentine. In the I_1_ tooth, Sr increases from the cusp into the vicinity of the cervical enamel and then establishes an oscillatory signature between local maxima and minima. Analysis of I_1_ and M_1–4_ from the same individual shown in Fig. 3a (illustrated with arrows) establishes that the I_1_ tooth concatenates the Sr variation within the molariform dentition. Alignment of M_1_ and M_2_ with the I_1_ record of the figured individual is more difficult than with M_3_ and M_4_ as the early gradational increase lacks the more discrete fluctuations that occur in the later-forming teeth.

### Other Macropodids

**Fig. 4:**
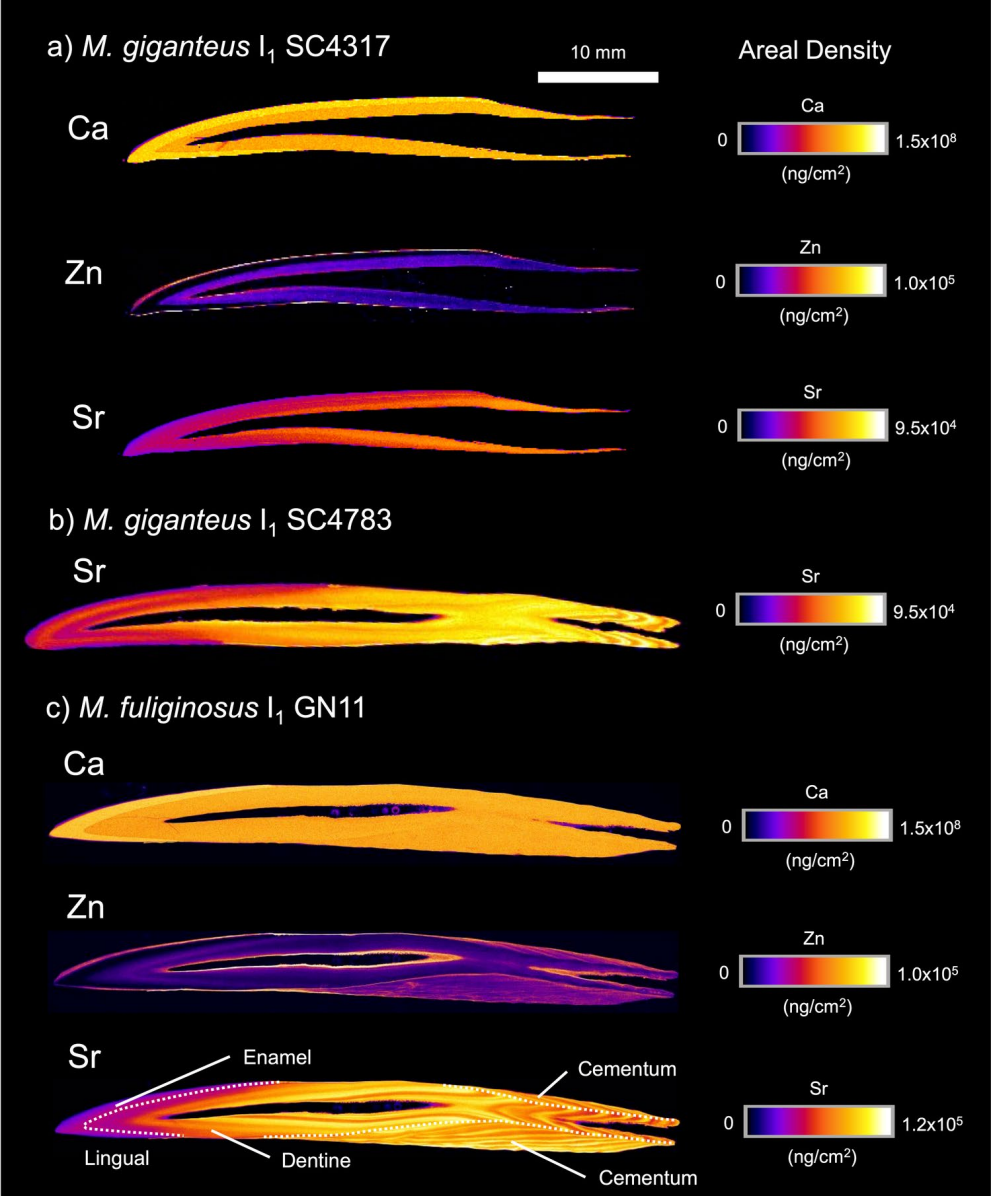
XFM maps of specified elements (Ca, Zn, and Sr) presented as areal density (ng/cm^2^) in the I_1_ tooth of two *M. giganteus* individuals (SC4317 and SC4783) and one *M. fuliginosus* individual (GN11). 10 mm scale bar is applicable to all teeth

Patterns of elemental incorporation observed in larger-bodied macropodids (Fig. 4) are broadly similar to those displayed in *N. eugenii* (Fig. 2). In older individuals, an oscillatory signal in Sr incorporation is clearly identified (Fig. 4b, c).

### *Trichosurus vulpecula*

**Fig. 5:**
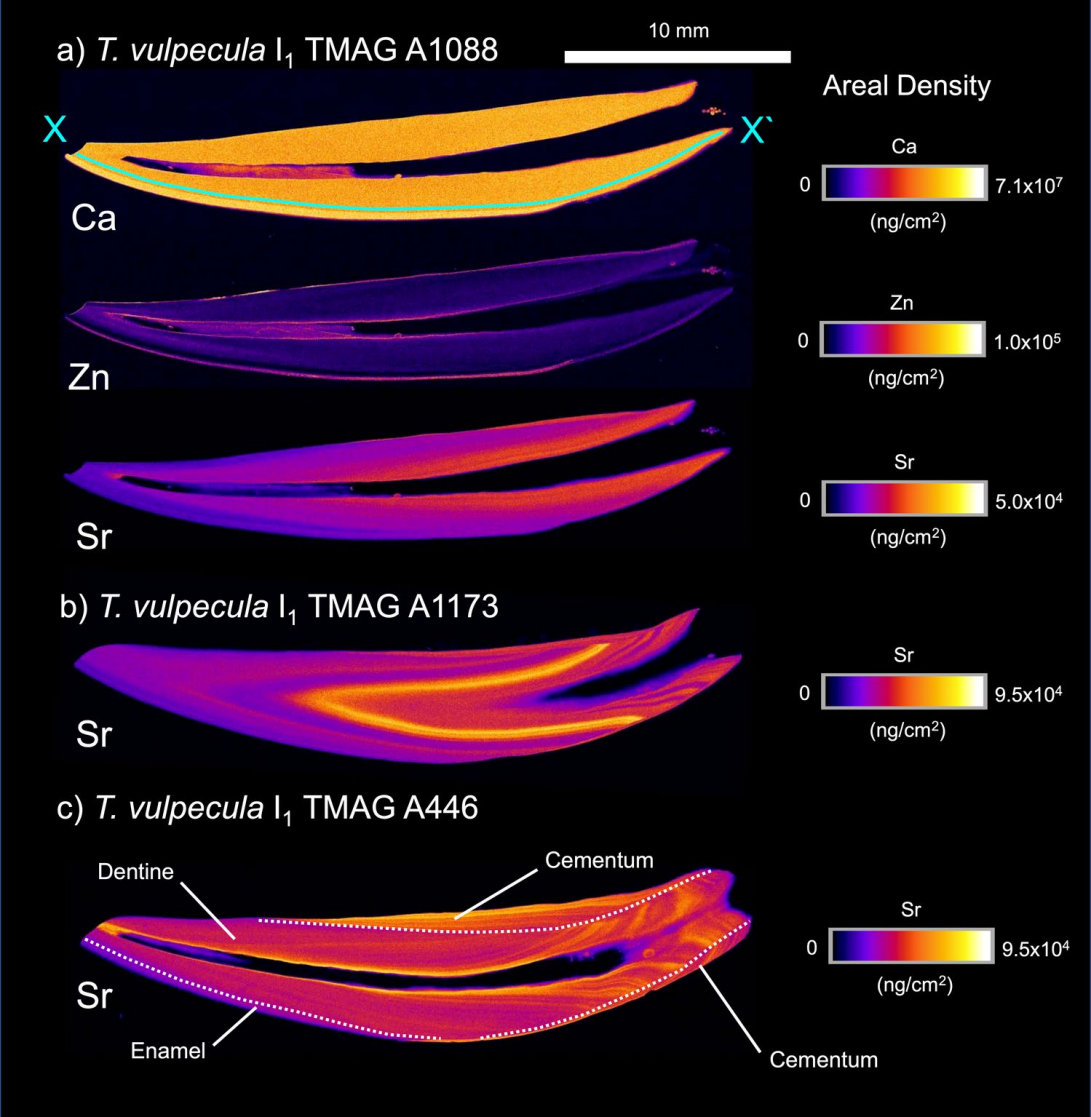
XFM maps of specified elements (Ca, Zn, and Sr) presented as areal density (ng/cm^2^) in the I_1_ tooth of three *T. vulpecula* individuals (TMAG A1088, TMAG A1173, and TMAGA446). The 10 mm scale bar is applicable to all teeth. TMAG A1088 is a sub-adult individual, with analysis of X-X` transect presented in Fig. 6

**Fig. 6:**
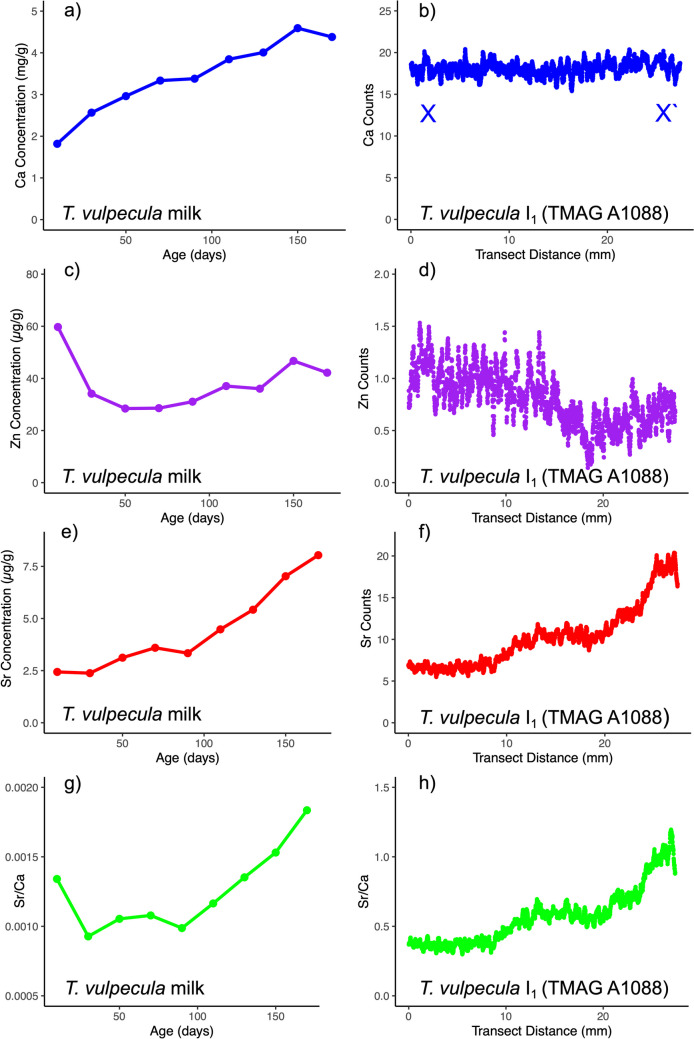
Comparison of milk and dentine composition (I_1_—TMAG A1088) for *T. vulpecula*. Milk composition—mean values for **a** Ca(mg/g), **c** Zn (µg/g), **e** Sr (µg/g), and **g** Sr/Ca in milk across lactation are presented. Age values (days) are the median of 20-day age classes. Data from [22]. Dentine composition—relative abundance (counts) for **b** Ca, **d** Zn, **f** Sr, and **h** Sr/Ca along the transect X-X` for individual TMAG A1088 (Fig. 5)

Within the examined incisors (Fig. 5), the distribution of Ca and Zn aligns with the overarching trends presented above. Similar to other examined taxa, Sr maps show a relatively low areal density at the cusp of the tooth (oriented on the left of Fig. 5) which increases towards the root. For comparative purposes, *T. vulpecula* milk composition across lactation for Ca, Zn, Sr, and Sr/Ca is depicted in Fig. 6 alongside recorded counts from the X-X` transect depicted in Fig. 5 for TMAG A1088. TMAG A1088 is a sub-adult individual aged between 1.5 and 2.5 years (Table 1). As such, the majority of tooth mineralisation in this individual can be associated with juvenile milk diet. In this individual, Sr areal density within dentine increases towards the base of the incisor, aligning with changes in milk composition seen in Fig. 6. Ca and Zn do not show similar alignment between dentine areal density and milk composition.

XFM analysis of two adult individuals (TMAG A1173 and TMAG A446) shows an oscillating signature in Sr that establishes in the later forming regions of the tooth. This signal is found in both cementum and dentine.

### *Vombatus ursinus*

**Fig. 7:**
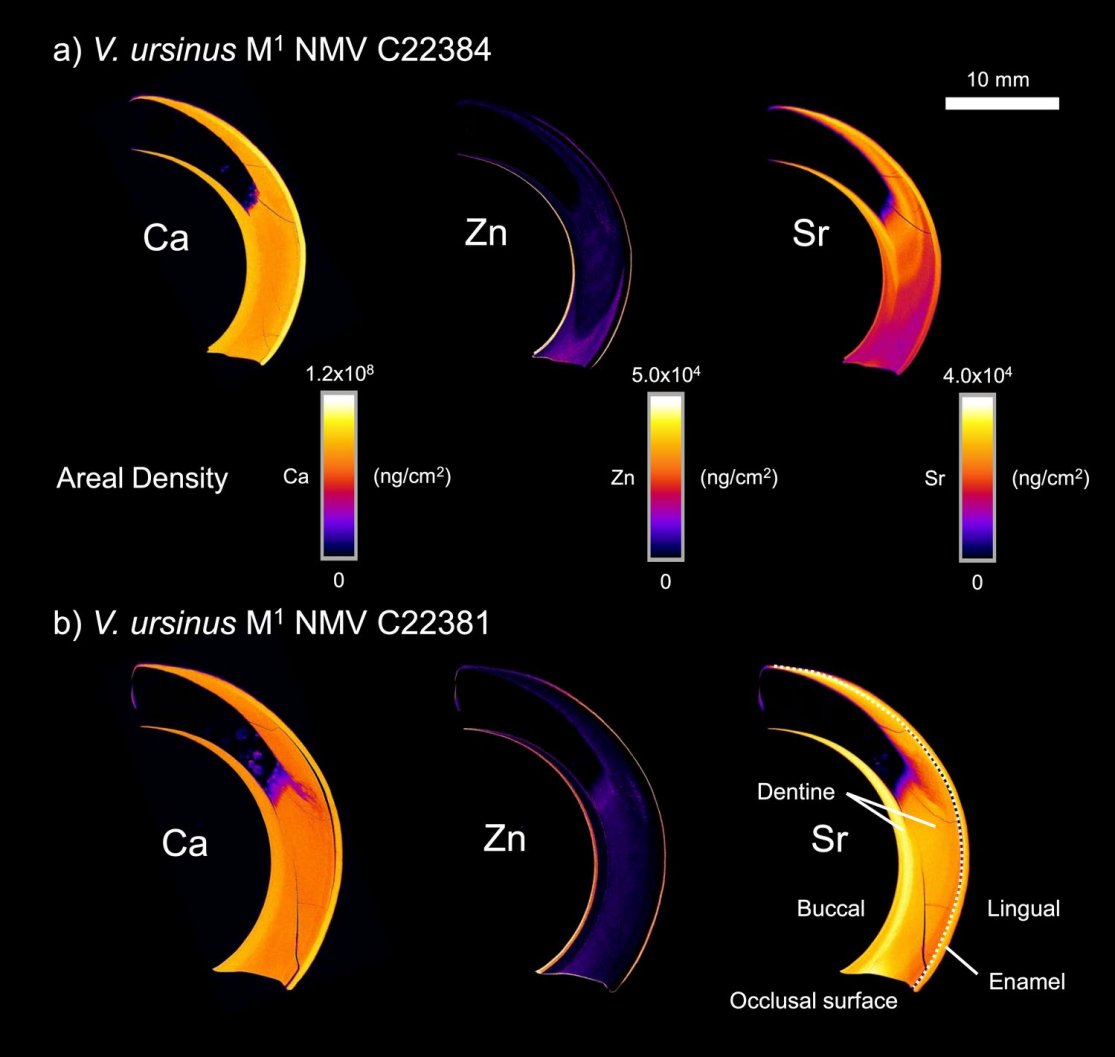
XFM maps of specified elements (Ca, Zn, and Sr) presented as areal density (ng/cm^2^) in the M^1^ tooth of two *V. ursinus* individuals (NMV C22384 and NMV C22381). 10 mm scale bar is applicable to all teeth

Across the examined individuals, the distribution of Ca within the upper first molar does not change between age classes. Unlike the other marsupials examined in this study, there is variation in Ca mineralisation within dentine (Fig. 7). The buccal portion of each upper tooth, which is not covered by enamel, shows an increased abundance of Ca compared to the centre of the dentine. Zn abundance is also high within the accreted cementum in this region and at the outer enamel surface. In the most juvenile individual examined, NMV C22384, the dentine near the occlusal surface shows an increase in Zn compared to the remainder of the tooth. In this same individual, Sr areal density increases markedly from the occlusal surface towards the root of the tooth, whereas in an older individual, the Sr signal is more oscillatory.

## Discussion

### Ca and Zn—Actively Incorporated Major and Trace Elements

Across the examined marsupial taxa, Ca and Zn are actively incorporated into the mineralising teeth. In enamel, Ca concentration is uniformly higher than in dentine and cementum, an expected finding due to the higher degree of enamel mineralisation [8]. In XFM analysis, both cementum and dentine show similar Ca incorporation with no distinct boundary between the tissues. Ca findings in the majority of the examined marsupials align with well-established patterns of enamel and dentine mineralisation in eutherian mammals. The ever-growing (euhypsodont) molars of *V. ursinus* are outliers when considering these broader patterns of Ca incorporation. On the buccal surface of the upper teeth, which is not covered by enamel, there is a section of dentine with higher Ca areal density. This is likely an adaptation to increase dentine hardness on the ‘knife-like’ leading edge of the ever-growing molariform dentition during chewing [31]. Among extant marsupials, this tooth form is unique to the three species of wombat and this variation in dentine has not been quantified in previous studies of their tooth material properties [32]. Our results suggest that there are further specialisations in the euhypsodont marsupial dentition beyond the continued formation of enamel and dentine.

Zinc is a trace element that fulfils numerous critical functions within the body and possesses noteworthy anti-microbial and anti-acid dissolution properties [33]. It is also fundamental to the mineralisation of enamel, dentine, and cementum [9]. Across the marsupials examined in this study, Zn abundance is highest at the outer enamel surface, in cementum and at the border of the pulp cavity. In hominoids, the concentration of Zn in the outer enamel surface may be a consequence of enamel mineralisation/maturation [9, 33, 34]. We find that this distribution of Zn is common to the teeth of marsupials and so may be a process fundamental to enamel formation. Zn is also associated with the neo-natal line in primates and annual banding in cementum [8, 9, 34]. Though no evidence of birth is found in the teeth of these marsupials, incremental lines in cellular cementum can be accentuated in XFM mapping of Zn (Figs. 2c and 4c). In the macropodids and *T. vulpecula*, this provides a secondary method to confirm periodicities observed in histological sections of cementum. We consider it more likely that these Zn bands are related to the pattern of cementum deposition but do not exclude some seasonal fluctuation in Zn uptake.

At present, the literature examining elemental incorporation within the teeth of primates is far more comprehensive than for other taxa. A number of features of Zn incorporation into marsupial teeth revealed in this study show similarities with these eutherian mammals. This is perhaps unexpected, as most marsupials possess both tubular enamel and dentine, with tubules that are continuous across the enamel-dentine junction. In the case of Zn, similarities between marsupial and primate teeth include an increase in abundance near the outer enamel surface (OES), higher incorporation in dentine than in enamel (barring the region adjacent to the OES), and a peak in Zn at the boundary of the pulp chamber [9, 33]. Cementum is also enriched in Zn in both marsupials and primates. These similarities give credence to the proposal that this distribution of Zn across the tooth may be partially adaptive, potentially playing a role in preventing erosion and calculus formation while also inhibiting cementum resorption [35]. This patterning of Zn incorporation is likely common to most mammals and may be ubiquitous in teeth.

### Sr—A Passively Incorporated Trace Element

Little fractionation in strontium was observed between enamel, dentine, and cementum in *T. vulpecula*, *M. giganteus*, *M. fuliginosus*, and *N. eugenii*. This illustrates that strontium incorporation into dental tissues is a passive process in these marsupials, with measured concentrations in a given mineralised tissue as a consequence of ingested uptake. This uptake occurs because of the similarities between Ca and other group 2 elements, notably Sr and Ba, facilitating substitution within the crystalline hydroxyapatite lattice [34]. The measured abundance of Sr within dental tissues is likely influenced by some level of discrimination against Sr by the digestive system. It has previously been suggested that the ability of the gut to discriminate against strontium increases across the lifespan in primates [36, 37], though more recent work has contradicted these findings [38]. A general trend of decreasing strontium abundance towards the base of the incisor root, seen in some individuals (e.g. Figure 3b, c), indicates potential for there to be some change in the ability of the gut to discriminate against Sr across the marsupial lifespan.

Sr uptake in *V. ursinus* dentine appears to vary between the regions with higher and lower Ca abundance. This suggests that Sr distribution in this situation may be a product of the ratio of Sr to Ca uptake, with more mineralised regions within the same tissue displaying a higher Sr areal density, but still capturing the same fluctuations in abundance.

### Indicators of Life History

In all tooth regions that form during pouch development, there is a progressive increase in Sr abundance. This is from low levels seen in the earliest mineralising enamel and dentine to a local maximum that generally occurs after the formation of the I_1_ tooth crown. Data from [22] presented in Fig. 6 suggest that this transition is indicative of changing milk composition during pouch development. This is an important finding as it indicates that Sr abundance is a viable indicator of changes in milk composition and the subsequent weaning transition to an adult diet. It remains to be determined if the main driver of this pattern is the absolute concentration of Sr or the ratio of Sr/Ca, as it is possible for these to vary independently. In the milk of *T. vulpecula* (Fig. 6), there is broad agreement in the trend of Sr concentration and Sr/Ca ratio, apart from at the earliest data point, which is likely prior to, or concurrent with, earliest tooth mineralisation. This makes it difficult to determine which of these factors is the driver of the observed patterns.

As well as being a method to reconstruct life history, compositional analysis of early-forming teeth also presents as a way to understand variation in passively incorporated trace elements within marsupial milk. This includes important environmental pollutants like lead and cadmium that can be maternally transferred through milk [39]. The method is less applicable to elements like Ca and Zn which are actively incorporated into mineralising teeth and show greater partitioning across enamel, dentine, and cementum. Ca concentration changes markedly in marsupial milk (Fig. 6, [22]), but this was not recorded in the level of mineralisation of enamel, dentine, or cementum. Of note is that, unlike the other marsupials in this study, the most juvenile individual of *V. ursinus* shows Zn enrichment in early-forming dentine (which we interpret as prior to the completion of weaning). Compared to Ca and Sr, the milk of *T. vulpecula* shows less change in Zn concentration over the period of lactation. The observed Zn pattern presents the testable hypothesis that there are key compositional differences in vombatid milk, or that this is instead a feature of the early development of the euhypsodont tooth growth form.

In the tooth root of adult individuals of all examined taxa, an oscillatory signature in Sr areal density is present, interpreted here as having a yearly periodicity. This seasonal variation in trace element incorporation has previously been identified in the teeth of eutherian mammals [e.g. 35, 40]. It is likely that environmental variation is impacting diet and/or soil particle/dust ingestion to influence the concentration of specific trace elements within mineralised tissues [40]. Further work is required to determine the driver of these patterns in the teeth of marsupials. The extended records of seasonality found in Sr areal density demonstrate that the procumbent lower incisor roots of *T. vulpecula*, *M. giganteus*, *M. fuliginosus*, and *N. eugenii* are ever-growing, elongating posteriorly within the jaw. This further establishes the pattern of ever-growing incisor roots in diprotodont marsupials beyond the species examined in [5].

### Broader Applicability to Living and Extinct Taxa

The vastness of continental Australia, along with cryptic behaviours, means that minimal data exists for the life history of many extant marsupials, particularly at population-specific resolutions. Our findings indicate that aspects of milk composition, weaning, and age can be examined using trace element profiles of the dentition. This can facilitate study of life history using museum specimens in cases where field observation may be extremely challenging.

This analysis provides the fundamental groundwork for the examination of life history in extinct marsupial taxa, which is particularly important for understanding marsupial physiology, evolution, and extinction events. Prior to the last ~ 100,000 years, the Australian marsupial community included far more megafaunal taxa, with the largest species being more than an order of magnitude larger than the marsupials alive today [41]. Establishing patterns of trace element incorporation in the teeth of living marsupials is an important step in reconstructing the development and weaning processes of extinct marsupial megafauna, complementing isotopic methods [27, 42–44]. Revealing these phenomena at species- and population-specific levels is fundamental to better understand the extinction of the largest marsupials [45].

### Conclusions

This study quantifies variation in the mineralisation of Ca, Sr, and Zn in the teeth of diprotodont marsupials during pouch development, weaning, and adulthood. Each of these elements represents a key chemical constituent of mineralised tissue. Ca is an actively incorporated major element, Zn is an actively incorporated trace element, and Sr is a passively incorporated trace element. As such, an understanding of the mobility of these three elements can inform patterns of elemental incorporation more broadly.

Synchrotron XFM highlights the utility of the lower diprotodontian incisor as a target for chemical analysis. Sr has good potential for examining life history patterns in marsupials, capturing milk composition through to weaning and a strong seasonal oscillatory signal in adult individuals. Trace element mapping has the potential to augment the data-deficient life histories of marsupials, living, and extinct.

## Supplementary Information

Below is the link to the electronic supplementary material.

Supplementary file1 (DOCX 16 KB)

## Data Availability

No datasets were generated or analysed during the current study.
