# Supplementary material for: Synchrotron X-ray Fluorescence Microscopy Reveals Trace Elemental Indicators of Life History in Marsupial Teeth
Source: Biol Trace Elem Res. 2025 Jan 16;203(9):4607–19. doi: 10.1007/s12011-024-04502-z (PMC12316728; doi:10.1007/s12011-024-04502-z)
Supplement: Supplementary file 1 — Supplementary file1 (DOCX 16 KB) [file 12011_2024_4502_MOESM1_ESM.docx]

| Species | Specimen number | Tooth type | Figure | Step size in X and Y (µm) | V_max  (mm/s) | Scan reference |
| --- | --- | --- | --- | --- | --- | --- |
| *Notamacropus eugenii* | 7024 | I_1_ | 1, 2, 3 | 100 | 5 | 24156 |
|  | 7024 | M_1_ | 1, 2, 3 | 10 | 2 | 24175 |
|  | 7024 | M_2_ | 1, 2, 3 | 10 | 2 | 24176 |
|  | 7024 | M_3_ | 1, 2, 3 | 10 | 2 | 24177 |
|  | 7024 | M_4_ | 1, 2, 3 | 10 | 2 | 24171 |
|  | 6623 | I_1_ | 1, 2, 3 | 10 | 10 | 130481 |
|  | 7140 | I_1_ | 1, 2, 3 | 10 | 10 | 130478 |
| *Macropus giganteus* | SC4317 | I_1_ | 4 | 100 | 10 | 24147 |
|  | SC4783 | I_1_ | 4 | 100 | 5 | 24218 |
| *Macropus fuliginosus* | GN11 | I_1_ | 4 | 10 | 10 | 130457 |
| *Trichosurus vulpecula* | TMAG A446 | I_1_ | 5, 6 | 10 | 10 | 130482 |
|  | TMAG A1088 | I_1_ | 5, 6 | 10 | 10 | 130484 |
|  | TMAG A1173 | I_1_ | 5, 6 | 10 | 10 | 130485 |
| *Vombatus ursinus* | NMV C22384 | M^1^ | 7 | 20 | 5 | 51740 |
|  | NMV C22381 | M^1^ | 7 | 20 | 5 | 51738 |

Supplementary Table 1. Scan parameters for teeth in this study analysed at the X-Ray Fluorescence Microscopy (XFM) beamline of the Australian Synchrotron. Mapping was undertaken with the Kirkpatrick-Baez microprobe and Maia detector at 18.5 KeV. Data available on request.
